# Machine Learning (ML) and Molecular Dynamics–Driven Optimization of VEGFR2 Ligands against Hepatocellular Carcinoma

**DOI:** 10.32604/or.2026.076072

**Published:** 2026-04-22

**Authors:** Farzana Yasmeen, Abdul Manan, Wook Kim, Sangdun Choi

**Affiliations:** 1 Department of Molecular Science and Technology, Ajou University, Suwon, Republic of Korea; 2 S&K Therapeutics, Suwon, Republic of Korea

**Keywords:** Hepatocellular carcinoma (HCC), machine learning, quantitative structure-activity relationship (QSAR), vascular endothelial growth factor receptor 2 (VEGFR2), RDKit, molecular docking and dynamics

## Abstract

**Objectives:**

Vascular endothelial growth factor receptor 2 (VEGFR2) is a critical therapeutic target in hepatocellular carcinoma (HCC) due to its role in angiogenesis and tumor progression. While several inhibitors are currently used, clinical utility is often limited by resistance and adverse effects, necessitating the discovery of novel therapeutic agents. The aim of this study was to identify and characterize novel, highly effective VEGFR2 inhibitors using an integrated computational pipeline to advance the development of new HCC treatments.

**Methods:**

A comprehensive dataset from the ChEMBL database was curated and standardized for Quantitative Structure-Activity Relationship (QSAR) modeling. A binary classification framework was employed, where a Light Gradient Boosting Machine (LGBM) model demonstrated superior predictive performance. Two lead compounds and a reference were selected for in-depth molecular modeling. Their binding poses were predicted via molecular docking and subsequently subjected to 200 ns Molecular Dynamics (MD) simulations to assess stability and conformational dynamics. Thermodynamic binding affinities were calculated using the Molecular Mechanics Poisson-Boltzmann Surface Area (MMPBSA) method.

**Results:**

The LGBM model achieved high accuracy and a robust Matthews Correlation Coefficient (MCC) on an independent test set. MD analysis, including Root Mean Square Deviation (RMSD) and Radius of Gyration (Rg), confirmed stable binding throughout the 200 ns trajectory. MMPBSA calculations validated the binding affinities, identifying van der Waals and electrostatic interactions as the primary driving forces for complex stability.

**Conclusion:**

This study successfully bridges machine learning with advanced molecular simulations, offering a validated workflow for the rational design and optimization of novel small-molecule VEGFR2 inhibitors.

## 1 Introduction

Hepatocellular carcinoma (HCC) is a primary malignancy of the liver and one of the most lethal cancers globally, with a rising incidence [1]. A hallmark of HCC progression is pathological angiogenesis, the formation of new blood vessels that supply the tumor with oxygen and nutrients, which is essential for its growth, metastasis, and survival [2]. The vascular endothelial growth factor (VEGF) pathway is a central regulator of this process [3,4]. Among the VEGF receptors, VEGF Receptor 2 (VEGFR2), also known as kinase insert domain receptor (KDR), is the primary mediator of VEGF-driven angiogenesis [5,6]. Its expression is significantly upregulated in HCC cells and tumor-associated endothelial cells compared to normal liver tissue, and high expression is correlated with a poor prognosis. The binding of the ligand VEGF-A to VEGFR2 triggers a cascade of downstream signaling events that promote endothelial cell proliferation, migration, and survival, making it a critical therapeutic target for combating HCC [7,8].

Targeting the VEGFR2 signaling pathway has emerged as a successful strategy in the systemic treatment of advanced HCC [9]. Small-molecule kinase inhibitors are a class of compounds designed to block the function of a specific protein, in this case, VEGFR2, by competitively binding to its ATP-binding site [10–13]. These inhibitors typically disrupt the phosphorylation cascade initiated by the receptor’s activation, thereby halting the pro-angiogenic signals. A prominent example is the multi-targeted kinase inhibitor sorafenib, which was the first systemic agent approved for advanced HCC [14,15]. While sorafenib primarily targets VEGFR2, it also inhibits other kinases like RAF and PDGFR, contributing to its broad anti-proliferative effects [16].

The success of sorafenib has paved the way for the development of newer, more selective small-molecule VEGFR2 inhibitors, such as lenvatinib, regorafenib, and cabozantinib, which have shown improved efficacy in clinical trials for HCC [17–19]. These agents are also multi-targeted but often possess different kinase inhibition profiles, allowing them to overcome some resistance mechanisms and provide alternative treatment options. For instance, regorafenib and cabozantinib are approved for second-line treatment in patients who have progressed on sorafenib. The continuous quest for more effective and safer small-molecule inhibitors underscores the importance of a deep understanding of the molecular interactions between these compounds and their target. Optimizing these interactions is key to improving selectivity and potency and reducing off-target effects, which are a major source of toxicity [17–19].

Despite the clinical success of these agents, a significant challenge remains: the majority of approved inhibitors are multi-targeted, leading to substantial off-target toxicities and a constrained therapeutic window. For example, the broad kinase profile of sorafenib contributes to adverse events such as hand-foot skin reaction and hypertension. Therefore, there is a pressing need to discover next-generation small-molecule inhibitors that possess enhanced selectivity for VEGFR2 while maintaining high potency, thereby potentially improving the therapeutic index and patient compliance. Our computational approach is designed to overcome this limitation by searching a chemical space for compounds possessing novel scaffolds that are distinct from the established urea- and quinoline-based chemotypes of current drugs, specifically optimizing for favorable predicted physicochemical and binding properties against the VEGFR2 active site. This targeted strategy is key to realizing the therapeutic potential of more selective agents.

The design and discovery of novel small-molecule inhibitors can be significantly accelerated by leveraging advanced computational methods [20–24]. Based on the current challenges in VEGFR2 inhibitor discovery, we hypothesize that a machine learning (ML)-based Quantitative Structure-Activity Relationship (QSAR) model, integrated with molecular docking and dynamic simulations, can identify novel and highly potent small-molecule inhibitors for the treatment of HCC. Accordingly, the aim of this study is to develop an ML-driven pipeline to predict the biological activity of new chemical entities, characterize their binding modes, and identify key structural features required for optimal VEGFR2 inhibition.

## 2 Material and Methods

**Figure 1:**
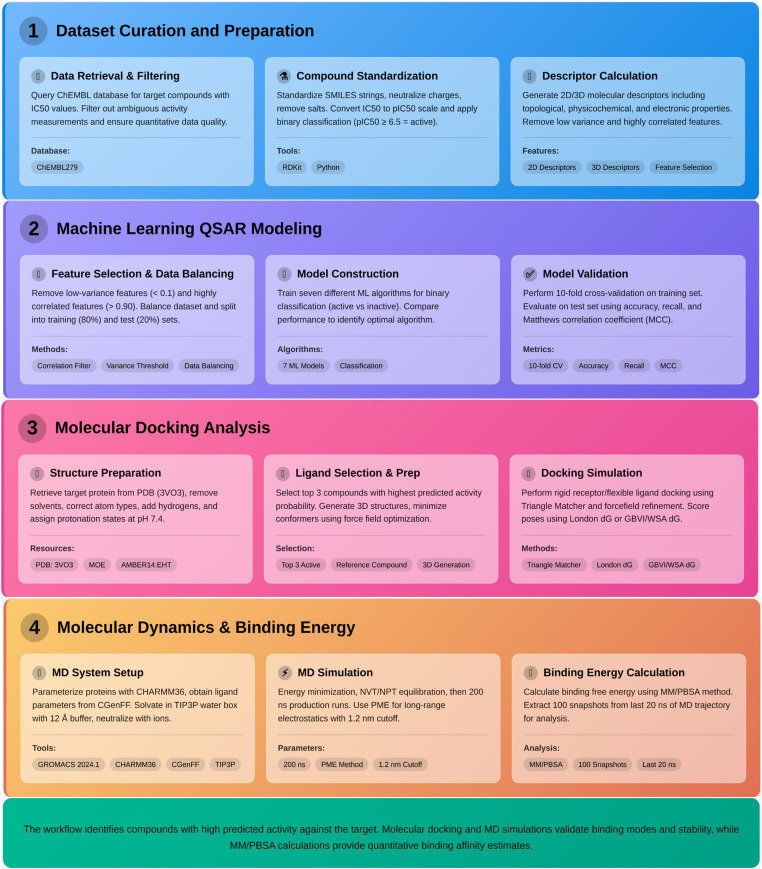
QSAR-Based Drug Discovery Workflow. Comprehensive pipeline for target-specific compound classification & optimization. Abb: MOE: Molecular Operating Environment, PDB: Protein Data Bank, EHT: Extended Hückel Theory, GBVI: Generalized Born Volume Integration (often used in solvation models), WSA: Water-Surface Area (related to solvation calculations), CGenFF: Charms General Force Field, NVT: Number of Particles, Volume, Temperature, NPT: Number of Particles, Pressure, Temperature, PME: Particle Mesh Ewald.

This section outlines the detailed computational and experimental procedures employed to develop a robust QSAR model for classifying active and inactive compounds against a target protein. The methodology integrates machine learning with molecular modeling techniques, providing a comprehensive *in silico* drug-discovery workflow (Fig. 1).

### 2.1 Dataset Curation and Preparation

#### 2.1.1 Data Retrieval and Filtering

A comprehensive dataset of compounds and their associated biological activity data retrieved from the ChEMBL database (version 36) (https://www.ebi.ac.uk/chembl/). The database was queried for compounds (ChEMBL279) with a defined target and a specified assay type (e.g., inhibition, antagonism). The query was refined to include only compounds with a reported half-maximal inhibitory concentration (IC_50_) value, ensuring quantitative activity data was available. All entries with ambiguous or poorly defined activity measurements (e.g., “active”, “>IC_50_”) were excluded. Following rigorous data cleaning and chemical standardization including duplicate structure removal, exclusion of inconsistent activity annotations, removal of missing values, and canonical SMILES standardization, the final curated dataset reduced to 10,016 unique compounds ensuring that each compound was represented only once with a single experimentally assigned bioactivity value. No ambiguity buffer or intermediate category applied. Duplicate compounds were eliminated using canonical SMILES matching following salt removal, charge neutralization, and molecular standardization using RDKit. Compounds associated with conflicting bioactivity labels across assays were excluded to prevent label ambiguity.

#### 2.1.2 Compound Standardization and Activity Classification

All retrieved compounds, represented by their SMILES strings, underwent a rigorous standardization process using the RDKit Python library. This includes neutralizing any charges, removing salts and mixtures, and canonicalizing the SMILES to ensure a consistent representation for each molecule. IC_50_ values were converted to their negative logarithmic form, pIC_50_, using the formula:

(1) $${\text{pIC}}_{50}=-{\text{log }}_{10}({\text{IC}}_{50}\times 10^{-6})$$

This transformation converts the data to a more normally distributed format and provides a more intuitive scale for biological activity.

For the QSAR-based classification problem, a strict cutoff value of pIC_50_ > 6.5 was applied to define the “active” class of compounds, while compounds with a pIC_50_ < 6.5 were labeled as “inactive”. This binary classification is a standard practice in QSAR modeling to create a clear separation between potent and non-potent compounds. The selection of pIC_50_ > 6.5 (corresponding to IC_50_ < 309 nM) as the threshold, for classifying compounds as active, was based on a combination of biological significance, pharmacological precedence, and statistical necessity for robust QSAR model development. This stricter threshold, which exceeded the common pIC_50_ = 6.0 minimum, ensured that only compounds exhibiting clear and significant inhibitory activity against VEGFR2 were included, thereby reducing the influence of borderline molecules. Furthermore, this cut-off was aligned with the reported potency of established clinical VEGFR2 inhibitors (such as Sunitinib and Sorafenib) and was consistent with common practice in kinase inhibitor QSAR studies. Finally, the 6.5 value empirically was optimized across our dataset to provide the best balance between maintaining a potency-focused definition of activity and avoiding severe class imbalance for effective machine learning analysis. As the dataset was already nearly balanced, no resampling techniques (e.g., SMOTE, random oversampling, random under sampling) were required (Table 1).

**Table 1:** The distribution of pIC_50_ activity and count of the dataset.

Class label	pIC_50_ range	Count (N)	Percentage (%)
Active	>6.5	5047	50.4
Inactive	<6.5	4969	49.6

Abb: pIC_50_: Negative log of the IC_50_ value.

#### 2.1.3 Molecular Descriptor Calculation

To represent the chemical structures in a format suitable for machine learning, a set of molecular descriptors were calculated for each compound. RDKit-based library (version 2025.09.3) used to generate a diverse array of 2D and 3D PubChem descriptors, including topological, physicochemical, and electronic properties. This process transformed the molecular structure into a numerical vector, which served as the input features for the machine learning models. Descriptors with low variance or those that were highly correlated removed to reduce redundancy and prevent overfitting.

### 2.2 Machine Learning-Based QSAR Modeling

#### 2.2.1 Feature Selection, Data Balancing, and Splitting

A rigorous feature selection procedure was conducted to refine the molecular descriptor set. A correlation-based filter method was employed, which systematically removed features that could compromise model performance. To rigorously prevent data leakage, each chemical entity was represented only once, and no compound appeared simultaneously in the training and test partitions across any experimental repeat. To reduce dimensional redundancy and noise, prior to model development, feature filtering was performed to enhance numerical stability and minimize PubChem descriptor redundancy. Specifically, low-variance features (variance < 0.1) eliminated to discard descriptors with minimal information content as such, features provide negligible discriminatory power for classification. Additionally, highly collinear features (correlation > 0.90) were removed to reduce redundancy and mitigate multicollinearity, thereby decreasing feature complexity and preventing overfitting. In each correlated pair, the descriptor with the lower univariate relevance toward the activity label was removed. These thresholds are widely adopted in QSAR modeling to improve model interpretability, reduce overfitting, and ensure numerical robustness. After feature selection, the dataset was randomly split into a training set (80%) and a test set (20%). This partition ensured that the model’s performance was evaluated on unseen data, providing an unbiased assessment of its predictive power. The training set used to train and optimize the machine learning models, while the test set held out for final performance validation.

#### 2.2.2 QSAR Model Construction and Validation

The study aimed to construct classification QSAR models to predict two bioactivity classes of VEGFR2 inhibitors: active, and inactive. A total of seven different machine learning algorithms were independently employed for model construction, allowing for an unbiased, and mechanistically interpretable comparative analysis to identify the best-performing algorithm. Naïve Bayes (probabilistic), Logistic Regression (linear), k-Nearest Neighbors (instance-based), Support Vector Classifier (kernel-based), Gaussian Process Classifier (Bayesian kernel-based), Gradient Boosting (ensemble tree-based), and Light Gradient Boosting Machine (boosted decision tree ensemble) were used. Naïve Bayes (NB) and Logistic Regression (LR) serve as baseline linear and probabilistic models. k-Nearest Neighbors (KNN) captures local similarity relationships aligned with classical QSAR assumptions. Support Vector Classifier (SVC) and Gaussian Process Classifier (GPC) model nonlinear decision boundaries via kernel learning. Gradient Boosting (GB) and LightGBM represent state-of-the-art ensemble learners capable of capturing high-order descriptor interactions. This diversity minimizes model-selection bias and ensures that predictive conclusions reflect intrinsic structure–activity relationships rather than algorithm-specific artifacts.

To achieve statistically robust and unbiased model evaluation, a 10× repeated stratified random resampling protocol was employed. In each repetition, the dataset split into 80% training and 20% independent testing sets, with stratification preserving the original active/inactive class distribution. Hyperparameter tuning was performed exclusively within the training set, and final model evaluation was conducted strictly on the corresponding independent test set. This entire procedure was repeated across ten independent random seeds, and all predictive performance metrics were reported as mean ± standard deviation (SD), thereby providing a reliable estimate of model stability and generalization rather than an optimistic single-split result. Hyperparameters were optimized using grid-search-based tuning, with the best-performing configuration per repetition selected based on AUC maximization. Model performance was assessed using multiple complementary metrics, including Accuracy, Precision, Recall, F1-score, Matthews Correlation Coefficient (MCC), and Area under the ROC curve (AUC) score. This multi-metric evaluation framework ensures comprehensive assessment across class balance, discriminative power, robustness, and predictive reliability. An external validation was also done to check the generalizability of the best model.

Unlike conventional QSAR studies that rely on a single random split, the present work implements a 10× repeated stratified resampling framework, which substantially reduces sampling bias, performance inflation, and random seed dependency. This validation strategy significantly strengthens the statistical reliability and regulatory robustness of the developed models and aligns with modern best-practice recommendations for machine learning in drug discovery.

### 2.3 Molecular Docking

Based on the QSAR model’s predictions, three of the most promising active compounds (i.e., compounds with the highest predicted probability of being active) were selected for further in-depth analysis. One of these compounds was a well-known reference compound. Molecular docking simulations performed using MOE (version 2020.09) by Chemical Computing Group to predict the binding pose and affinity of these ligands to the target protein.

*Protein and Ligand Preparation*

The 3D structure of the target protein retrieved from the Protein Data Bank (PDB) https://www.rcsb.org/ (PDB ID: 3VO3). The protein was loaded into MOE and subjected to the structure preparation protocol. This process involves the removal of solvent molecules and extraneous ions, the correction of atom types, and the addition of explicit hydrogen atoms. The protonation states of ionizable residues (e.g., histidine) assigned based on their microenvironments and a physiological pH of 7.4. Finally, a force field, such as AMBER14: EHT, was applied to the protein structure.

The selected ligands were prepared within MOE. Their 3D structures generated from their 2D representations, and their conformers minimized using the same force field applied to the protein. This step ensures that the ligands are in their lowest energy state prior to docking by scoring function.

### 2.4 Docking Simulation

The docking simulation was performed using MOE’s Dock application. A rigid receptor and flexible ligand approach was employed. The binding pocket was defined by identifying the cavity around a co-crystallized (PDB ID: 3VO3) ligand. Prior to docking, the protein and the ligands were prepared with MOE; this preparation involved the removal of the co-crystallized ligand, as well as any water molecules, salts, and other extraneous compounds from the VGFR2 structure to expose the active site. The docking algorithm was set to a high-resolution mode, such as the Triangle Matcher followed by force field refinement, which generated multiple binding poses by matching ligand features to receptor features and then optimized these poses using a force field.

The Amber14: EHT force field was implemented for all energy minimization and refinement steps. The EHT (Extended Hückel Theory) correction applied specifically to the ligand parameters to improve the accuracy of the partial charge assignments. The binding pocket for subsequent docking and flexible refinement was defined strictly based on the co-crystallized Type II inhibitor found in the 3VO3 PDB structure, having coordinates (*X* = 25.61, *Y* = −27.70, *Z* = −13.52). Prior to screening, the reliability of the docking protocol was confirmed by re-docking the co-crystallized ligand. The predicted pose was scored and compared to the experimental crystal structure. The calculated root mean square deviation (RMSD) was 1.78 Å, confirming the accuracy of the protocol in reproducing the known binding conformation (typically, an RMSD ≤ 2.0 Å is acceptable).

The poses were scored using a scoring function, such as the London dG or GBVI/WSA dG, which estimates the free energy of binding. The top-scoring poses for each ligand were retained for further analysis. The poses with the lowest binding energy (i.e., the most negative score) were visually inspected to confirm favorable interactions with key active site residues and selected for subsequent molecular dynamics (MD) simulations.

### 2.5 MD Simulation

To assess the stability of the protein-ligand complexes and to refine the binding energy, MD simulations were performed for the three selected complexes (two compounds from the QSAR model and the reference compound), using GROMACS 2024.1 [25]. The systems were carefully prepared by parameterizing the proteins with the CHARMM36 all-atom force field, while ligand parameters were obtained through the CGenFF server. Each complex was solvated in a cubic water box with a 12 Å buffer using TIP3P water molecules [26], followed by neutralization with Na^+^ and Cl^−^ ions. Energy minimization successfully removed unfavorable steric clashes, leading to a relaxed system. Subsequent equilibration stabilized both temperature (300 K) and pressure (1 bar), first under NVT conditions with a Nose–Hoover thermostat (1 ps) and then under NPT conditions with a Parrinello–Rahman barostat (5 ps) [27]. The systems were then subjected to long production runs of 200 ns, during which stable trajectories were obtained with a trajectory snapshot collection frequency of every 10 ps (10,000 integration steps). Short-range electrostatic and van der Waals interactions were treated with a 1.2 nm cutoff, whereas long-range electrostatics were accurately handled by the Particle Mesh Ewald method [28]. Post-simulation analyses revealed that the protein–ligand complexes maintained structural integrity throughout the simulation, providing a reliable basis for assessing their dynamic behavior and interaction stability. MD simulations were carried out to validate the stability of the final complexes [29].

### 2.6 Binding Free Energy Calculation

The Molecular Mechanics/Poisson-Boltzmann Surface Area (MMPBSA) method was applied to the MD trajectories to calculate the binding free energy (ΔG_bind_) for each complex [30,31]. This end-state method provided a more accurate estimation of binding affinity than simple docking scores by accounting for the desolvation and entropic effects. The binding free energy calculated using the following equation:

(2) $$\Delta {\text{G}}_{\text{bind}}=\Delta {\text{E}}_{\text{MM}}+\Delta {\text{G}}_{\text{solvation}}-\text{T}\Delta \text{S}$$

where, ΔE_MM_ is the change in molecular mechanics energy, ΔG_solvation_ is the change in solvation free energy, and TΔS is the change in conformational entropy. The trajectory snapshots are used to calculate these terms, providing a final, more reliable ranking of the selected compounds based on their thermodynamic stability within the binding site. In this study, the conformational entropy term (T∆S) was not calculated based on two factors: T∆S calculation, typically performed via Normal Mode Analysis (NMA) or quasi-harmonic analysis, is highly computationally demanding for large biological systems and long trajectories. The resulting entropy values often have large associated errors and high variance, potentially outweighing their contribution to the overall binding energy, particularly when comparing similar compounds in a binding pocket. Therefore, the reported ∆G_bind_ values are the sum of the molecular mechanics and solvation energy terms (excluding entropy). We primarily used these values for relative ranking and comparison of the binding affinities of the selected compounds.

## 3 Results

### 3.1 Exploratory Data Analysis (EDA)

**Figure 2:**
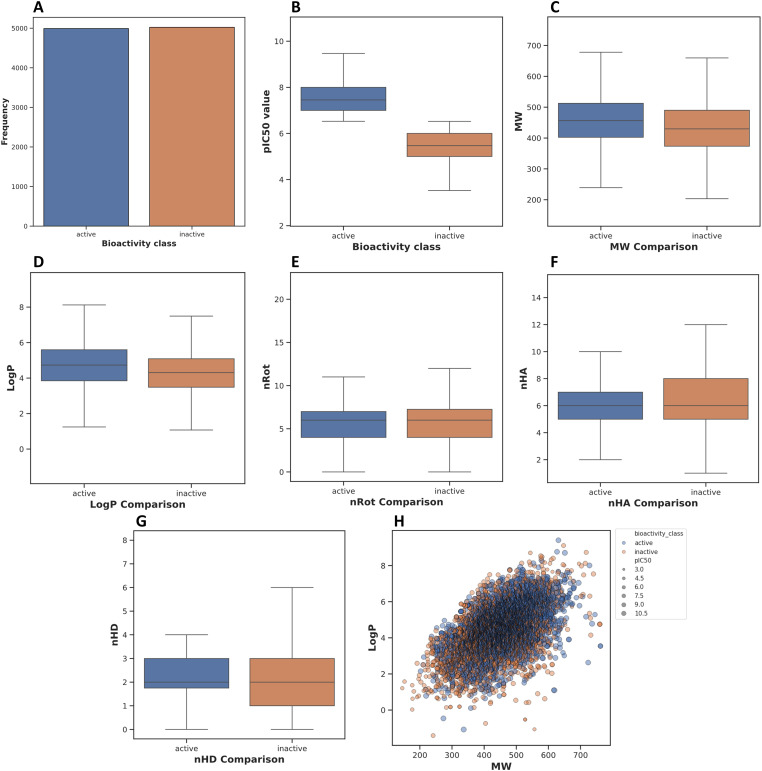
Box plots comparing key physicochemical properties between active and inactive compounds. The active compounds were defined as having a pIC_50_ value greater than 6.5. (**A**): Frequency count of compounds classified as active vs. inactive in the dataset. (**B**): Distribution of pIC_50_ values. (**C**): Comparison of molecular weight (MW) distribution. (**D**): Comparison of the LogP (octanol-water partition coefficient) distribution. (**E**): Distribution of the number of rotatable bonds (nRot). (**F**): Distribution of the number of hydrogen bond acceptors (nHA). (**G**): Distribution of the number of hydrogen bond donors (nHD). (**H**): Scatter plot of LogP vs. molecular weight (MW), with points colored by bioactivity class and sized according to their pIC_50_ value.

The EDA provided key insights into the physicochemical property distributions of the active and inactive compound classes (Fig. 2; Table 2). The data were visualized using box plots to compare the distributions of pIC_50_, molecular weight (MW), calculated octanol-water partition coefficient (logP), and the number of rotatable bonds (nRot). The distribution of the dataset confirms that the data collection is relatively balanced with approximately equal numbers of compounds classified as active and inactive. This ensures that the subsequent machine learning models are trained on a non-skewed target variable distribution (Fig. 2A).

- **pIC**_**50**_ **Distribution**: As expected from the data classification criteria, a clear separation was observed between the active and inactive classes based on their pIC_50_ values. The median pIC_50_ for the active class was significantly higher at approximately 6.5, with the interquartile range (IQR) spanning from roughly 6.2 to 7.0. In stark contrast, the median pIC_50_ for the inactive class was substantially lower, centered at approximately 4.5. This distinct separation validates the binary classification scheme (Fig. 2B).
- **Molecular Weight (MW) Distribution**: The median molecular weight of the active compounds (approximately 400 Da) was slightly higher than that of the inactive compounds (approximately 380 Da). The box plot for the active class showed a broader distribution, with a larger IQR, indicating a greater diversity in molecular size among the active compounds. Notably, both classes predominantly conform to Lipinski’s Rule of Five, with the majority of compounds having a molecular weight below 500 Da (Fig. 2C).
- **Lipophilicity (logP) Distribution**: The logP values, a measure of lipophilicity, showed a modest difference between the two classes. The median logP for the active compounds (approximately 3.5) was slightly higher than the median for the inactive compounds (approximately 3.0). The active class also displayed a wider spread in logP values, as evidenced by a larger IQR, suggesting that a range of lipophilicities can be tolerated for activity (Fig. 2D).
- **Conformational Flexibility & Hydrogen Bonds**: The number of rotatable bonds (nRot), indicated a nearly identical distribution for both groups (Fig. 2E), with most compounds possessing between 3 and 7 rotatable bonds, suggesting comparable structural rigidity. Similarly, the distributions for hydrogen bond features were highly overlapping. The number of hydrogen bond acceptors (nHA) primarily ranged from 4 to 8 (Fig. 2F), and the number of hydrogen bond donors (nHD) clustered between 1 and 3 for both active and inactive molecules (Fig. 2G), suggesting that these specific properties are not primary determinants for bioactivity class differentiation in this dataset.
- **MW vs. LogP:** A bivariate scatter plot of LogP vs. MW, with point color indicating bioactivity and size reflecting pIC_50_. While the two classes broadly overlap in the chemical space defined by these two properties, the most potent compounds (largest, darkest blue circles) tend to reside in the moderate LogP and MW range of the cluster (Fig. 2H).

**Table 2:** Exploratory data analysis (EDA) of a small molecule dataset comparison between VEGFR2 active and inactive compounds.

	Property	MW	LogP	H-Accept	H-Donors	Rotatable	TPSA
**Active class**	**Min**	202.242	−1.0763	1	0	0	17.3
	**Max**	761.91	9.407	15	8	17	241.66
	**50%**	456.546	4.73221	6	2	6	93.405
	**Mean**	457.4504	4.677768	6.036659	2.282051	5.896635	93.47301
	**Skewness**	−0.02365	−0.2982	0.22431	0.33086	0.516218	0.245628
	**Kurtosis**	−0.04723	0.009409	0.219619	0.224981	0.328327	0.814292
	***p*-value**	3.46 × 10^−55^	7.55 × 10^−52^	1.70 × 10^−05^	1.36 × 10^−72^	8.90 × 10^−02^	2.43 × 10^−30^
**Inactive class**	**Min**	145.165	−1.4125	1	0	0	13.22
	**Max**	759.934	9.1126	14	7	22	229.93
	**50%**	429.831	4.3041	6	2	6	87.06
	**Mean**	431.5902	4.292122	6.197452	1.895701	5.809912	87.94961
	**Skewness**	0.120903	−0.0677	0.084962	0.299466	0.51821	0.288969
	**Kurtosis**	0.001475	0.485405	−0.25196	0.032594	0.286897	1.054921
	***p*-value**	3.46 × 10^−55^	7.55 × 10^−52^	1.70 × 10^−05^	1.36 × 10^−72^	8.90 × 10^−02^	2.43 × 10^−30^

Abb: MW: Molecular Weight; LogP: Lipophilicity; H-Accept/Donors: Hydrogen Bond Acceptor/Donors; TPSA: Topological Polar Surface Area.

### 3.2 QSAR Model Performance

The performance of seven different machine-learning algorithms evaluated on the QSAR classification task using a balanced dataset. Among all evaluated models, LGBM exhibited the strongest overall performance, achieving the highest values across nearly all key metrics, including Accuracy (~0.83), F1-score (~0.82), MCC (~0.65), and AUC (~0.91), followed by SVC and GB, both of which demonstrated consistently high discriminative ability with AUC values approaching 0.89–0.90 and MCC values exceeding 0.63. The GPC showed strong competitive performance, outperforming KNN and LR across all major metrics, particularly in terms of Precision (~0.81), F1-score (~0.80), and AUC (~0.88), confirming its suitability for modeling nonlinear structure–activity relationships (Table 3). The KNN classifier demonstrated robust Recall (~0.82) and high AUC (~0.88), indicating effective sensitivity toward active VEGFR2 inhibitors, albeit with slightly reduced Precision and MCC compared with ensemble-based models (Supplementary Table S1). In contrast, the LR and NB models displayed substantially weaker performance. LR exhibited moderate Accuracy (~0.69) and MCC (~0.37), while NB performed the poorest overall, with Accuracy near 0.61, MCC around 0.22, and AUC below 0.70, indicating limited capacity to capture the complex nonlinear bioactivity landscape governing VEGFR2 inhibition. Representative best-performing hyperparameters of seven models shown in Supplementary Table S2.

**Table 3:** Summary of evaluation metrics of classification algorithms.

Models	Accuracy test	MCC test	AUC score
Naïve bayes (NB)	0.611 ± 0.005	0.224 ± 0.010	0.678 ± 0.009
Logistic regression (LR)	0.687 ± 0.005	0.375 ± 0.009	0.756 ± 0.005
K-Nearest neighbors (KNN)	0.799 ± 0.012	0.598 ± 0.025	0.880 ± 0.006
Gaussian process (GP)	0.803 ± 0.006	0.607 ± 0.012	0.883 ± 0.004
Gradient boosting (GB)	0.818 ± 0.006	0.636 ± 0.013	0.896 ± 0.004
Support vector classifier (SVC)	0.821 ± 0.007	0.643 ± 0.014	0.888 ± 0.004
Light gradient boosting machine (LGBM)	0.823 ± 0.005	0.647 ± 0.011	0.901 ± 0.004

Abb: MCC: Matthews Correlation Coefficient; AUC: Area under the Curve.

**Figure 3:**
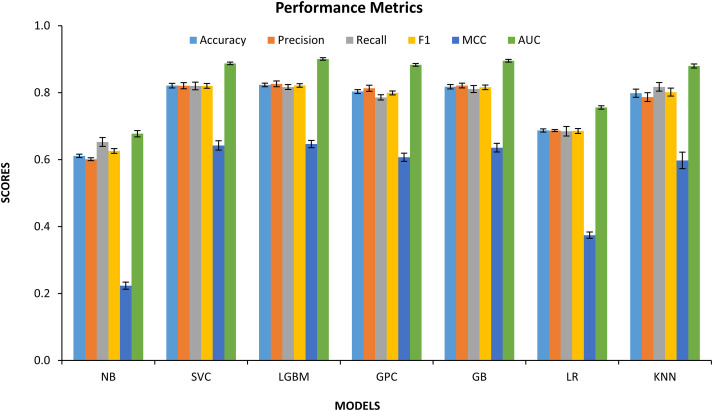
Comparative performance of seven machine learning classifiers for VEGFR2 inhibitor classification under a 10× repeated stratified random resampling protocol. Error bars denote the mean ± standard deviation, reflecting model stability and generalization robustness across repeated data partitions. Abb: NB: Naive Bayes, SVC: Support Vector Classifier, LGBM: Light Gradient Boosting Machine, GPC: Gaussian Process Classification, GB: Gradient Boosting, LR: Logistic Regression, KNN: K-Nearest Neighbors.

Fig. 3 presents a comprehensive comparison of seven machine learning classifiers such as Naïve Bayes (NB), Support Vector Classifier (SVC), Light Gradient Boosting Machine (LGBM), Gaussian Process Classifier (GPC), Gradient Boosting (GB), Logistic Regression (LR), and k-Nearest Neighbors (KNN) evaluated using Accuracy, Precision, Recall, F1-score, MCC, and AUC. Importantly, all high-performing models exhibit tightly clustered error bars, reflecting excellent stability across the 10 repeated resampling experiments and confirming that the reported performances were not driven by favorable random splits. The small standard deviations observed across repetitions confirm model stability rather than overfitting behavior.

The confusion matrix of the best-performing LGBM classifier, averaged across 10 repeated stratified test splits, revealed a strong balance between sensitivity and specificity (Supplementary Table S3). On average, 817.6 active compounds were correctly classified as active (true positives), while 832.3 inactive compounds were correctly predicted as inactive (true negatives). Misclassifications were limited, with 172.7 false positives and 181.4 false negatives, confirming that the high AUC and MCC values are not driven by class bias but reflect genuine bidirectional predictive power.

Moreover, to assess the model’s generalizability, an external validation set comprising 1438 compounds was employed. The LGBM model was tested on this independent dataset and achieved an accuracy of 0.604, with a sensitivity of 0.599 and a specificity of 0.610, demonstrating balanced and unbiased classification of active and inactive compounds.

### 3.3 Molecular Docking Simulation

**Figure 4:**
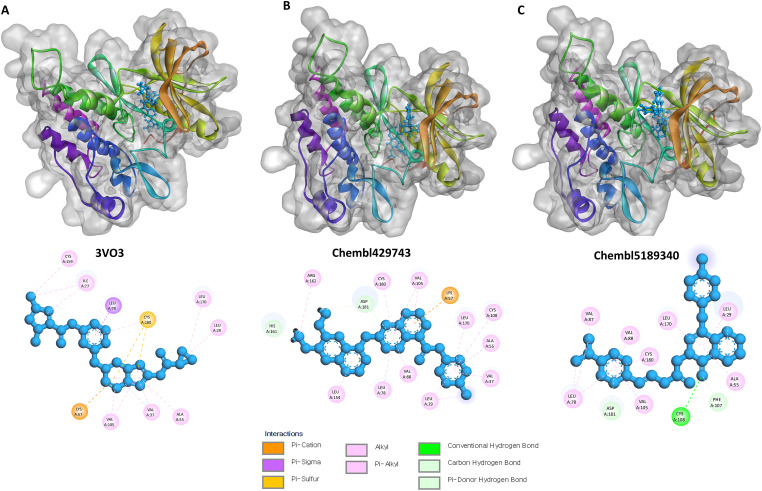
Three- and two-dimensional illustrations by Discovery Studio (version 2021) Visualizer. (**A**): The binding of ligand 3VO3 is characterized by an extensive network of hydrophobic interactions, including multiple alkyl interactions with residues like Val37 and Leu29, and further stabilized by cation/sulfur/sigma interactions. (**B**): The stabilization of the ChEMBL429743 complex is dominated by strong C-H bond interactions with His161 and Asp181, along with a vast network of alkyl interactions and a single cation interaction, while lacking conventional hydrogen bonds. (**C**): The ChEMBL5189340 ligand is anchored by a single, crucial hydrogen bond with the backbone of Cys108, with its overall stability significantly enhanced by C-H bonds and a robust series of alkyl interactions.

Molecular docking simulations were performed to predict the binding modes and key interactions of the three selected ligands, 3VO3 (−8.56003 kcal/mol; pIC_50_ = 9.022), ChEMBL429743 (−8.5322 kcal/mol; pIC_50_ = 10.522), and ChEMBL5189340 (−8.2495 kcal/mol; pIC_50_ = 10.638), within the protein’s active site. The analysis focused on identifying non-covalent interactions, including hydrogen bonds, C-H bonds, π-alkyl, π-cation, π-sulfur, and π-sigma interactions (Fig. 4; Table 4).

- **3VO3 Complex**: The ligand’s binding is primarily stabilized by an extensive network of hydrophobic interactions. The docking pose revealed multiple π-alkyl interactions with key residues, including Val37, Leu29, Ala55, Ile77, Val105, Cys159, and Leu170. Additionally, π-cation/sulfur/sigma interactions with Lys57, Cys180, and Leu78 further stabilized the complex. No conventional hydrogen bonds or C-H bonds were observed for this ligand in the most favorable pose (Fig. 4A).
- **ChEMBL429743 Complex**: This compound’s binding is dominated by strong C-H bond interactions with His161 and Asp181. A vast network of π-alkyl interactions with residues Leu29, Val37, Ala55, Leu78, Val88, Val105, Cys108, Leu154, Arg162, Leu170, and Cys180 also contributed significantly to its stability. A single π-cation interaction with Lys57 was also identified. No conventional hydrogen bonds were found (Fig. 4B).
- **ChEMBL5189340 Complex**: This ligand formed a single, crucial hydrogen bond with the backbone of Cys108. This strong, specific interaction appears to anchor the molecule within the active site. The binding is further stabilized by C-H bonds with Phe107 and Asp181. A robust series of π-alkyl interactions with Leu29, Ala55, Leu78, Val87, Val88, Val105, Leu170, and Cys180 also contributed to its stability. Unlike the other two ligands, no π-cation, π-sulfur, or π-sigma interactions are observed (Fig. 4C).

**Table 4:** Docked interaction analysis of VEGFR2 protein with ligands.

Compounds	H-Bonds	CH-Bond	π-Alkyl	π-Cation/Sulfur/Sigma
**3VO3** (−8.56003 kcal/mol; pIC_50_ = 9.022)	NA	NA	Val37, Leu29, Ala55, Ile77, Val105, Cys159, Leu170,	Lys57, Cys180, Leu78
**ChEMBL429743** (−8.5322 kcal/mol; pIC_50_ = 10.522)	NA	His161, Asp181	Leu29, Val37, Ala55, Leu78, Val88, Val105, Cys108, Leu154, Arg162, Leu170, Cys180,	Lys57
**ChEMBL5189340** (−8.2495 kcal/mol; pIC_50_ = 10.638)	Cys108	Phe107, Asp181	Leu29, Ala55, Leu78, Val87, Val88, Val105, Leu170, Cys180	NA

Abb: NA: not applicable.

### 3.4 Molecular Dynamics Simulation Analysis

**Figure 5:**
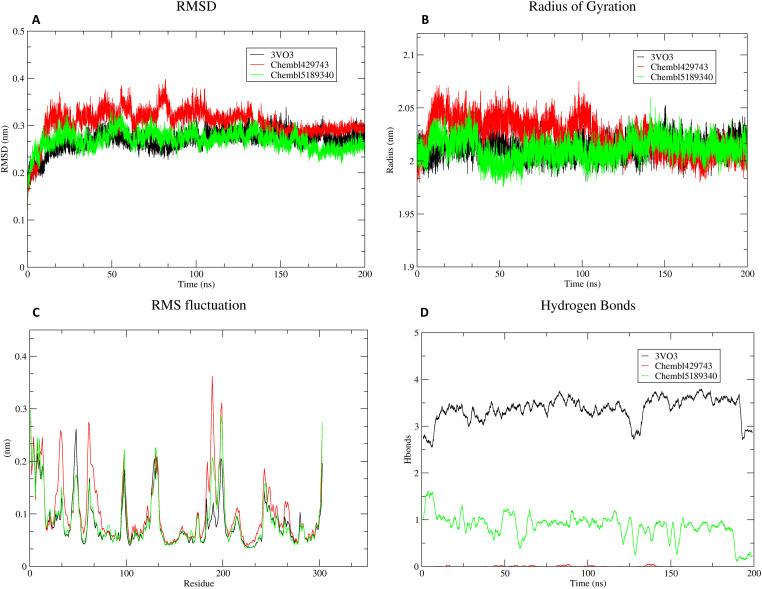
MD simulation of compounds during 200 ns time. The changes in the plots reveal the protein-ligand complex stability, flexibility, compactness, and interaction patterns. (**A**): RMSD (**B**): Radius of Gyration (**C**): RMS Fluctuation (**D**): Hydrogen Bonds.

MD simulations were performed for 200 ns to evaluate the stability and conformational dynamics of the protein in complex with three different ligands: 3VO3, ChEMBL429743, and ChEMBL5189340. The results analyzed are based on key metrics: Root Mean Square Deviation (RMSD), Radius of Gyration (Rg), Root Mean Square Fluctuation (RMSF), and the number of hydrogen bonds (Fig. 5).

- **RMSD and System Stability**: The RMSD plot of the protein backbone reveals that all three systems achieved stability after an initial equilibration period of approximately 20–30 ns. The black line (3VO3) and the green line (ChEMBL5189340) show stable RMSD values, fluctuating around an average of 0.25 nm and 0.28 nm, respectively. The red line (ChEMBL429743) exhibits slightly higher fluctuations but remains stable within a range of 0.25–0.4 nm. The consistently low RMSD values for all complexes indicate that the protein-ligand complexes maintain their overall structural integrity and are stable throughout the 200 ns simulation (Fig. 5A).
- **Radius of Gyration (Rg) and Protein Compactness**: The Rg plot indicates the overall compactness of the protein. The Rg values for all three complexes are stable, fluctuating around a mean of 2.0 nm for the black and green lines and slightly higher for the red line. The relative stability of Rg for all three systems, particularly after the initial 50 ns, confirms that the protein has reached a stable, folded conformation and the systems are well equilibrated. Significant unfolding events were not observed in any of the trajectories (Fig. 5B).
- **RMSF and Local Flexibility**: The RMSF plot highlights the fluctuations of individual protein residues. All three complexes exhibit similar fluctuation profiles, with the lowest RMSF values in the core helical regions, indicating high rigidity. The highest fluctuations are observed at the N- and C-termini and in loop regions (e.g., around residues 100–120 and 180–200). These fluctuations are characteristic of flexible regions of the protein that are typically exposed to the solvent and not involved in the core-binding site. The overall low RMSF values across most of the protein confirm a high degree of structural stability (Fig. 5C).
- **Hydrogen Bonds**: The number of hydrogen bonds between the ligand and the protein receptor was tracked over the simulation. The black line (3VO3) consistently forms a high number of hydrogen bonds, averaging between 3 and 4, indicating strong and stable interactions. In contrast, the green line (ChEMBL5189340) and the red line (ChEMBL429743) show significantly fewer hydrogen bonds, typically fluctuating between 0 and 2. This suggests that the binding of 3VO3 is primarily driven by strong, specific hydrogen bonding, while the binding of the other two ligands may be more reliant on hydrophobic or other non-covalent interactions (Fig. 5D).

### 3.5 Binding Free Energy Analysis (MMPBSA)

**Figure 6:**
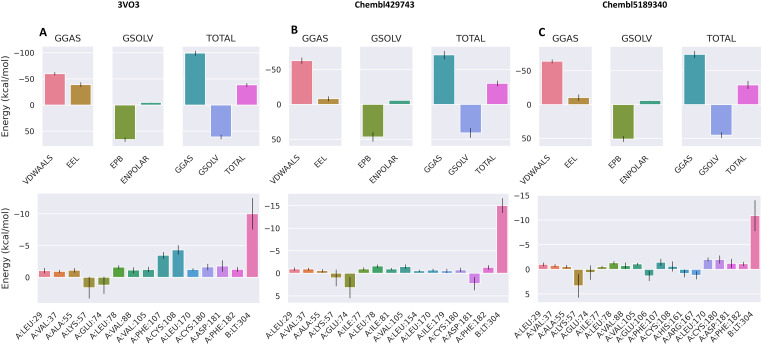
MMPBSA analysis. Binding free energy contribution by various interactions (top), binding free energy contribution by active residues and ligand (below). B:LT-304 indicates relevant small molecule inhibitor. (**A**): 3VO3 (**B**): ChEMBL429743 (**C**): ChEMBL5189340.

The binding free energy (ΔG_bind_) of the three protein-ligand complexes (3VO3 [Fig. 6A], ChEMBL429743 [Fig. 6B], and ChEMBL5189340 [Fig. 6C]) was calculated using the Molecular Mechanics/Poisson-Boltzmann Surface Area (MMPBSA) method on snapshots from the MD trajectories. The total binding free energy for each complex and the energy contributions of key residues were analyzed.

- **Overall Binding Free Energy**: As shown in the bar charts, all three ligands exhibited favorable (negative) total binding free energies, indicating stable binding to the protein. The total binding free energies were as follows: 3VO3 (−38.66 kcal/mol), ChEMBL429743 (−30.19 kcal/mol), and ChEMBL5189340 (−29.01 kcal/mol). This places the ligands in the following order of binding affinity: 3VO3 > ChEMBL429743 > ChEMBL5189340.
- **Energy Contributions**: The binding free energy is a composite of several terms. The gas-phase energy (GGAS), which includes van der Waals (VDWAALS) and electrostatic (EEL) interactions, was the main favorable contributor for all complexes. The solvation energy (G_SOLV_), including polar (EPB) and non-polar (ENPOLAR) terms, was unfavorable and opposed binding, as expected, due to the energy cost of desolvating the protein and ligand.
- **Residue-Specific Contributions**: The per-residue energy decomposition plots provide granular insight into the binding hot spots. For all three ligands, several common residues contributed favorably to the total binding energy, including A:LEU-29, A:GLU-74, and A:PHE-182. Notably, a specific residue, B:LT-304, showed a substantial favorable contribution only for the 3VO3 and ChEMBL429743 complexes but an unfavorable contribution for the ChEMBL5189340 complex. This suggests a unique interaction between these ligands and that residue.

## 4 Discussion

The binding and activity of small molecule inhibitors against a protein target are complex phenomena driven by a combination of chemical and biological factors. The analyses conducted in this study provide a comprehensive view of these interactions, focusing on VEGFR2, a key receptor tyrosine kinase implicated in angiogenesis and a validated target for cancer therapy. The machine-learning model’s ability to predict activity based on molecular descriptors suggests that specific physicochemical properties are critical for potent VEGFR2 inhibition. For instance, the observed trends of slightly higher molecular weight and lipophilicity in active compounds may be linked to the need for a larger scaffold to occupy the ATP-binding pocket of VEGFR2 and form extensive non-covalent interactions [32]. The lower number of rotatable bonds in active molecules points to the entropic advantage of a more rigid structure, which reduces the energetic cost of conformational changes upon binding to the relatively rigid active site of VEGFR2. These findings are consistent with established principles of medicinal chemistry and provide a rational basis for designing molecules that are predisposed to be active against this target.

The final selection of the two lead compounds, CHEMBL5189340 and CHEMBL429743, was a strategic process that filtered the rank-ordered QSAR hits based on a critical balance between predicted potency and optimal drug-like properties. Both compounds were chosen because they exhibited exceptionally high predicted activity (pIC_50_ values of 10.638 and 10.523, respectively), placing them at the top of the virtual screening list. Critically, they also satisfied a favorable physicochemical profile, adhering to key ADMET criteria like Lipinski’s Rule of Five, with appropriate molecular weights (MW < 500) and acceptable TPSA values (approx. 70–80) typical of oral small-molecule drugs. A strategic decision was made to select two compounds with a range of lipophilicity to explore the structure-activity landscape. CHEMBL5189340 possesses an optimal LogP of 3.857, favorable for absorption, while CHEMBL429743 has a higher, more lipophilic LogP of 7.103. This compound was deliberately retained to investigate whether its potentially superior binding interactions, a feature later supported by MD simulations, could justify the potential trade-offs associated with increased lipophilicity and subsequent formulation challenges.

The MD and MMPBSA analyses provided a dynamic, atomic-level understanding of the protein-ligand complexes that underpins their biological relevance. The strong and stable binding of the 3VO3 reference inhibitor, primarily through hydrophobic interactions and favorable gas-phase energies, align, with its known high potency against VEGFR2. This highlights the importance of a well-packed and desolvated hydrophobic core in the binding pocket for strong affinity. Conversely, the specific hydrogen bond observed for ChEMBL5189340 with Cys108 provides a key structural anchor, which is a common feature in Type II kinase inhibitors that bind to the DFG-out conformation [33,34]. This level of detail is crucial for explaining the observed differences in binding free energy and can guide the design of next-generation inhibitors with improved potency and selectivity. By integrating machine learning-based prediction with sophisticated molecular modeling and energy calculations, this study not only identifies potential therapeutic candidates but also provides the foundational biological and structural insights required for rational drug development against the VEGFR2 target, a critical step in the fight against cancer.

The observed discrepancy in predicted binding affinity between the rigid docking protocol and the MD simulation fundamentally arises from the difference between static and dynamic representations of the protein-ligand interaction. The docking protocol, which assumes a rigid protein structure (3VO3), uses an empirical scoring function that prioritizes the lowest energy pose within this fixed environment, often favoring extensive hydrophobic and pi-interactions, even if the geometry for a key hydrogen bond is slightly sub-optimal due to minor backbone deviations. Conversely, the MD simulation accounts for conformational flexibility, allowing the side chains and, crucially, the backbone residues of the hinge region to move and relax. The MD analysis demonstrated that the protein-ligand complex dynamically settles into a more stable, lower-energy conformation where the ligand’s key hydrogen bond donors and acceptors can achieve optimal alignment with the hinge region’s backbone atoms. This dynamic stabilization is essential for realizing the compound’s true, high-affinity binding and explains why the MD simulation provides a more accurate representation of the binding mode than the static docking result. The 3–4 hydrogen bonds consistently observed during the stable MD phase are formed predominantly with the backbone amide and carbonyl groups (Supplementary Fig. S1). The conventional protein-ligand hydrogen bonds that the docking scoring function failed to detect are due to the initial rigid-body constraints. This confirms that the primary stabilizing force of the reference compound is indeed strong, specific hydrogen bonding, captured by the dynamic nature of MD simulation.

The analysis of the lead compound, ChEMBL5189340, reveals a Type I binding mode, offering a crucial mechanistic distinction from the reference structure 3VO3, which is characteristic of Type II inhibitors. Specifically, the Type II mode seen in 3VO3 requires the inactive DFG-out conformation and extension into the adjacent allosteric pocket, while our MD simulations confirm that ChEMBL5189340 maintains the DFG-in conformation and anchors firmly within the ATP site through a single, defining hydrogen bond to the Cys108 hinge backbone. This is highly significant because, despite employing the DFG-out pocket (3VO3) as a template, our ML/MD pipeline successfully identified a potent hinge-binder, demonstrating its capacity to select compounds with mechanistically novel profiles. This Type I binding mechanism suggests that ChEMBL5189340 targets the active or apo state of VEGFR2, potentially offering an alternative therapeutic strategy to circumvent acquired resistance often associated with established multi-targeted Type II kinase inhibitors like Sorafenib and Regorafenib.

The EDA reveals crucial differences in the physicochemical properties of active and inactive compounds, which are fundamental to developing a robust QSAR model. The observed separation in pIC_50_ values confirms that the initial data classification is well defined and serves as a reliable foundation for building a machine-learning classifier. The distinct separation between the two classes is a strong indicator of the presence of a meaningful structure-activity relationship within the dataset.

The subtle but significant differences in molecular properties, including MW, logP, and nRot, between the active and inactive sets highlight key structural features that are likely essential for biological activity. The marginally higher median MW and logP in the active class, while adhering to the general principles of Lipinski’s Rule of Five, indicate a slight trend toward larger and more lipophilic molecules being more potent. This is attributed to the need for a larger molecular scaffold to form extensive van der Waals interactions with the target protein or to overcome desolvation penalties in the binding pocket. Conversely, the slightly lower number of rotatable bonds in the active class suggests that increased molecular rigidity may be advantageous for binding. A more rigid molecule has fewer degrees of freedom to lose upon binding, potentially leading to a more favorable entropic contribution to the binding free energy. This observation is in line with the “lock-and-key” model of molecular recognition, where a pre-organized, less flexible ligand is more likely to fit into a specific receptor site.

Overall, the trends identified in the EDA provide a solid rationale for the selection of molecular descriptors to train the QSAR model. The physicochemical properties examined here, along with other calculated descriptors, served as the input features for the machine-learning model. The distinct differences observed between the two classes suggest that a machine-learning algorithm should be able to discriminate effectively between active and inactive compounds based on their molecular properties. This initial analysis is a critical step in a cheminformatics pipeline, as it not only validates the data classification but also provides a mechanistic and structural basis for the QSAR model’s predictive power.

A marked performance gap between linear/probabilistic models and nonlinear learning algorithms was observed. LR and NB yielded substantially inferior predictive performance compared with ensemble and kernel-based methods such as LGBM, SVC, and GB. This pronounced disparity directly indicates that the VEGFR2 structure-activity landscape is fundamentally nonlinear and driven by high-order physicochemical interactions that cannot be captured by linear decision boundaries or conditional independence assumptions inherent to LR and NB. Linear models approximate activity as an additive combination of descriptors, while NB additionally assumes descriptor independence; both assumptions are violated in kinase inhibition, where cooperative steric, electronic, and hydrophobic effects govern binding. In contrast, the superior performance of LGBM, SVC, and GB demonstrates the necessity of nonlinear decision surfaces, adaptive feature weighting, and hierarchical feature interactions to accurately model VEGFR2 inhibition. These results therefore provide strong mechanistic justification for the use of nonlinear and ensemble-based learning strategies in VEGFR2-targeted QSAR modeling, rather than linear classifiers.

The LGBM model emerges as the optimal framework, delivering the highest accuracy, F1, MCC, and AUC values simultaneously. This indicates not only excellent class discrimination but also balanced sensitivity and specificity, a crucial requirement for real-world virtual screening where both false positives and false negatives carry high experimental costs. The highest MCC values observed for LGBM (~0.65), SVC (~0.64), and GB (~0.64) confirm that these models exhibit strong bidirectional predictive reliability across both active and inactive classes. Similarly, AUC values exceeding 0.88 for LGBM, SVC, GB, GPC, and KNN establish that these models provide excellent ranking capability, which is essential for lead prioritization in large-scale VEGFR2 inhibitor screening pipelines.

The MD simulation results provide a detailed dynamic view of the protein-ligand complexes, complementing the static insights from molecular docking. The stability observed in the RMSD and Rg plots across all three systems is a crucial finding. The quick attainment of a stable plateau, particularly after the initial 20–30 ns, confirms that the systems are well equilibrated and the selected docking poses are stable and representative of the protein’s native binding state. This stability is a strong indicator of a favorable protein-ligand binding conformation. The RMSF plot further deepens our understanding of the dynamic behavior of the protein. The high fluctuations in loop regions and termini are expected that do not compromise the stability of the binding site itself. Conversely, the low fluctuations in the core structure and binding pocket residues indicate that these regions are rigid and well packed, which is essential for maintaining the integrity of the binding site. The similar fluctuation patterns across the three complexes suggest that ligand binding does not induce significant large-scale conformational changes in the protein backbone.

A key point of discussion emerges from the hydrogen bond analysis. The consistently high number of hydrogen bonds formed by the 3VO3 complex suggests that these interactions are the primary drivers of its high affinity and stable binding. In contrast, the low number of hydrogen bonds for ChEMBL429743 and ChEMBL5189340 implies that their binding is likely dominated by other forces, such as hydrophobic interactions and van der Waals forces. This difference in binding mechanisms could explain potential differences in their binding free energies and overall potency, which is further investigated with MMPBSA calculations. These findings underscore the importance of dynamic simulations to capture the full picture of ligand-receptor interactions, which are often oversimplified in static docking poses. The observed differences in binding stability and interaction types between the ligands provide a strong basis for explaining their varied biological activities. The MMPBSA analysis provides a more thermodynamically rigorous assessment of binding affinity compared to simple docking scores, integrating the dynamic effects and conformational changes captured during the MD simulation. The calculated total binding free energies directly rank the three compounds in order of their predicted binding strength, with 3VO3 showing the strongest affinity, followed by ChEMBL5189340 and ChEMBL429743. This ranking is consistent with the stability observed in the RMSD analysis, where the 3VO3 complex displayed consistently stable interactions.

The decomposition of the binding free energy into its constituent parts is particularly informative. The large favorable contribution from gas-phase energy (GGAS) highlights the critical role of direct interactions like van der Waals and electrostatics in stabilizing the protein-ligand complex. This reinforces the importance of a good steric and electrostatic fit within the binding pocket. Conversely, the unfavorable solvation energy term underscores the energetic penalty associated with removing the water molecules from the binding interface, which is a key barrier to successful binding. The balance between these opposing forces dictates the overall binding affinity. This suggests that the differences in the chemical structure of the ligands lead to distinct modes of interaction with this specific residue, which likely contributes significantly to the observed differences in their binding affinities. This type of detailed analysis is invaluable for guiding future ligand optimization by providing a clear focus on which specific interactions to enhance or modify to improve binding. Despite the robust nature of ML-MD pipelines, this study has certain limitations. First, while the QSAR and MD results are theoretically sound, the identified lead compounds require further experimental validation through *in vitro* kinase assays and cell-based studies to confirm their biological efficacy. Second, the MMPBSA calculations omitted the entropic term, which, while standard for relative ranking, may affect the absolute binding free energy values. Finally, the study focused on a limited number of lead compounds; a broader exploration of the chemical space and potential off-target interactions with other kinases will be necessary to fully establish the clinical potential of these candidates.

## 5 Conclusion

This study successfully employed an integrated computational approach to identify and characterize potent small-molecule inhibitors of the target protein. The initial EDA revealed key physicochemical differences between active and inactive compounds, providing a strong rationale for the subsequent machine learning-based QSAR modeling. Ensemble and kernel-based learning strategies represent the state-of-the-art for VEGFR2 inhibitor QSAR modeling. Among all tested models, LGBM stands out as the most powerful and reliable predictive framework, closely followed by SVC and GB, while linear and probabilistic models exhibit limited applicability. Subsequent molecular docking and MD simulations provided a detailed mechanistic understanding of the binding of three selected compounds. While all three complexes showed structural stability over the 200 ns MD simulation, the analyses revealed distinct binding mechanisms.

The 3VO3 complex stabilized predominantly by extensive π-alkyl and π-cation interactions, while ChEMBL5189340 relied on a crucial anchoring hydrogen bond with Cys108. The final MMPBSA calculations confirmed these findings, with 3VO3 exhibiting the most favorable total binding free energy, primarily driven by strong gas-phase electrostatic and van der Waals interactions. The residue-level energy decomposition provided a high-resolution view of binding hotspots, highlighting common and unique interaction profiles for each ligand. This multi-faceted analysis not only validated the predictive power of the QSAR model but also provided crucial insights into the key non-covalent interactions essential for high-affinity binding, which was leveraged for future rational drug design efforts to optimize lead compounds.

The integrated approach successfully identified two lead compounds, ChEMBL429743 and ChEMBL5189340, which exhibited superior predicted potencies and favorable drug-like properties. Rigorous MD simulations further confirmed their stable binding modes and significant contributions from key energetic terms. While the calculated binding free energies (∆G_bind_) intended only for relative ranking due to the omission of the entropic term, their highly favorable values confirm the substantial stability of the predicted complexes.

## Supplementary Materials



## Data Availability

Data available on request from the authors. The data that support the findings of this study are available at https://github.com/abdulmanan012/VEGFR2.

## Supplementary Materials

The supplementary material is available online at https://www.techscience.com/doi/10.32604/or.2025.076072/s1.
