# Supplementary material for: Machine Learning (ML) and Molecular Dynamics–Driven Optimization of VEGFR2 Ligands against Hepatocellular Carcinoma
Source: Oncol Res. 2026 Apr 22;34(5):24. doi: 10.32604/or.2026.076072 (PMC13126583; doi:10.32604/or.2026.076072)
Supplement: Supplementary file 1 [file OncolRes-34-76072-s001.docx]

**Machine Learning (ML) and Molecular Dynamics–Driven Optimization of VEGFR2 Ligands against Hepatocellular Carcinoma**

**Farzana Yasmeen^1a^, Abdul Manan^1a*^, Wook Kim^1^, Sangdun Choi^1,2*^**

^1^Department of Molecular Science and Technology, Ajou University, Suwon, 16499, Korea

^2^S&K Therapeutics, Suwon 16502, Republic of Korea

^*^Corresponding Author Email: [mananriaz012@gmail.com](mailto:mananriaz012@gmail.com); [sangdunchoi@ajou.ac.kr](mailto:sangdunchoi@ajou.ac.kr)

^a^These two authors contributed equally.

**Supplementary Table S1.** Performance metrics among seven models on test set after 10x random resampling protocol.

| **Models** | **Accuracy** | **Precision** | **Recall** | **F1** | **MCC** | **AUC** |
| --- | --- | --- | --- | --- | --- | --- |
| **NB** | 0.611 | 0.601 | 0.653 | 0.626 | 0.224 | 0.678 |
| **SVC** | 0.821 | 0.821 | 0.820 | 0.821 | 0.643 | 0.888 |
| **LGBM** | 0.823 | 0.827 | 0.817 | 0.822 | 0.647 | 0.901 |
| **GPC** | 0.803 | 0.813 | 0.786 | 0.799 | 0.607 | 0.883 |
| **GB** | 0.818 | 0.821 | 0.811 | 0.816 | 0.636 | 0.896 |
| **LR** | 0.687 | 0.687 | 0.685 | 0.686 | 0.375 | 0.756 |
| **KNN** | 0.799 | 0.787 | 0.818 | 0.802 | 0.598 | 0.880 |

Abb: F1: F-score; MCC: Matthews correlation coefficient; AUC: Area Under the Curve; NB: Naïve Bayes; SVC: Support Vector Classifier; LGBM: Light Gradient-Boosting Machine; GPC: Gaussian Process Classification; GB: Gradient Boosting; LR: Logistic Regression; KNN: K-Nearest Neighbors

**Supplementary Table S2.** Representative best-performing hyperparameters of seven models

| **Models** | **Hyperparameter tuning** |
| --- | --- |
| **SVC** | C = 10, gamma = scale, kernel = RBF |
| **LGBM** | learning_rate = 0.1, max_depth = 10, n_estimators = 200, num_leaves = 50 |
| **GB** | learning_rate = 0.1, max_depth = 5, n_estimators = 200, subsample = 0.8 |
| **GPC** | RBF kernel, max_iteration = 100 |
| **KNN** | RBF kernel, max_iteration = 100 |
| **LR** | L2 regularization, lbfgs solver |
| **NB** | - |

Abb: RBF: Radial Basis Function

**Supplementary Table S3.** Confusion matrix of the optimized LGBM classifier across 10x repeated stratified test splits.

| **Confusion matrix** | **Predicted Inactive** | **Predicted Active** |
| --- | --- | --- |
| **Actual Inactive** | 832.3 (TN) | 172.7 (FP) |
| **Actual Active** | 181.4 (FN) | 817.6 (TP) |

Abb: TN: True Negative, FN: False Negative, FP: False Positive, TP: True Positive.


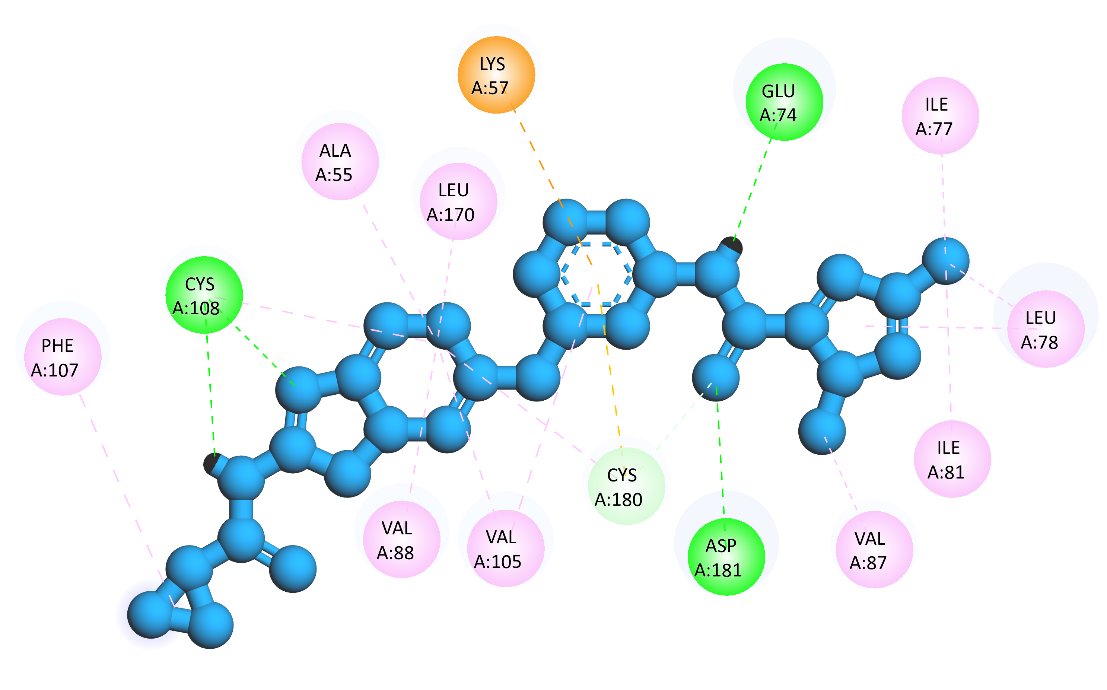


**Supplementary Figure S1:** Protein-ligand complex of 3VO3 at 100 ns, showing hydrogen bonds (green)
